# Real-World Efficacy of Bevacizumab in Patients With Recurrent Epithelial Ovarian Cancer

**DOI:** 10.3389/fonc.2022.843278

**Published:** 2022-05-19

**Authors:** Jo-Ni Hung, Shih-Tien Hsu, Lou Sun, Sheau-Feng Hwang, Chih-Ku Liu, Yu-Hsiang Shih, Ming-Jer Chen, Jun-Sing Wang, Chien-Hsing Lu

**Affiliations:** ^1^ Department of Obstetrics and Gynecology, Taichung Veterans General Hospital, Taichung, Taiwan; ^2^ Center for General Education, Ling Tung University, Taichung, Taiwan; ^3^ School of Medicine, China Medical University, Taichung, Taiwan; ^4^ Department of OB/GYN, School of Medicine, National Yang Ming Chiao Tung University, Taipei, Taiwan; ^5^ Department of Medicine, School of Medicine, National Yang Ming Chiao Tung University, Taipei, Taiwan; ^6^ Division of Endocrinology and Metabolism, Department of Internal Medicine, Taichung Veterans General Hospital, Taichung, Taiwan; ^7^ College of Medicine, National Chung-Hsing University, Taichung, Taiwan; ^8^ Ph.D. Program in Translational Medicine, Institute of Biomedical Sciences, National Chung-Hsing University, Taichung, Taiwan; ^9^ Rong-Hsing Research Center for Translational Medicine, National Chung-Hsing University, Taichung, Taiwan

**Keywords:** recurrent epithelial ovarian cancer, bevacizumab (Avastin^®^), real-world study, progression, survival outcome

## Abstract

**Background:**

Bevacizumab in combination with chemotherapy prolonged the progression-free survival (PFS) of patients with recurrent epithelial ovarian cancer (EOC) in large-scale randomized controlled trials. However, real-world data for the use of bevacizumab in Asian patients with EOC is lacking. This study investigated the efficacy of adding bevacizumab to chemotherapy and compared it with that of chemotherapy alone in patients with recurrent EOC using real-world data from an Asian population.

**Method:**

We conducted a retrospective cohort study using data from a tertiary medical center in central Taiwan. Patients who had EOC with first relapse between 2011 and 2019 were enrolled. Patients’ medical histories, medication treatment, and relevant information were collected. The outcomes were PFS and overall survival (OS). The Kaplan-Meier plot was used to generate a survival curve for OS and PFS. Cox proportional hazard analysis was used to determine the associations of Bevacizumab treatment with OS and PFS with adjustment of relevant variables. Subgroup analyses were conducted to determine if there was a significant variation in the aforementioned associations.

**Results:**

After a median follow-up of 23 months, 67% of patients in the Bevacizumab group and 81% of patients in the non-Bevacizumab group had disease progression or death. There was no significant between-group difference in OS (p = 0.475). The median duration of PFS was 18.9 and 9.6 months, respectively, favoring those who were treated with Bevacizumab. After multivariate adjustment, treatment with Bevacizumab was associated with a lower risk of disease progression (hazard ratio 0.33, 95% CI 0.13-0.85, p = 0.021). The improvement in PFS was consistent in the subgroups of different histological types, different disease stages at diagnosis, different treatment-free intervals, those undergoing or not undergoing secondary cytoreductive surgery, diverse chemotherapy regimens.

**Conclusion:**

Our findings provided crucial insights into the efficacy of bevacizumab for the treatment of recurrent EOC in the real-world setting.

## Introduction

Epithelial ovarian cancer (EOC) is the second most common and the most lethal gynecological cancer in developed countries (1). Most of the patients are diagnosed as having advanced stage disease and experience relapse of the disease despite achieving complete remission. Approximately 49% of patients die within 5 years (2). Resistance to chemotherapy is commonly observed in patients with recurrent EOC, and its treatment is considerably challenging. Therefore, the prognosis of recurrent EOC is poor, with a progression-free survival (PFS) of approximately 8–13 months (2–4). Asian countries had the largest number of incident cases (~171,000) and mortality (~113,000) of EOC in 2020 (5), and only a small proportion of Asian patients (<30%) were enrolled in previous clinical trials (2, 3).

Bevacizumab, a humanized monoclonal antibody against angiogenesis, has been approved by the US Food and Drug Administration for the treatment of recurrent EOC since 2016 (6–10). Bevacizumab in combination with chemotherapy prolonged the PFS of patients with primary (6, 8) or recurrent (7, 9) EOC in large-scale randomized controlled trials (RCTs). However, patient enrollment into RCTs is usually highly selective, and most of them are from Western countries. Moreover, differences in the histological types and prognosis of EOC between Asian and Caucasian patients have been reported (11). Therefore, real-world data for the use of bevacizumab in Asian patients with EOC (1, 12) are required, especially for those with recurrent EOC.

This study investigated the efficacy of adding bevacizumab to chemotherapy and compared it with that of chemotherapy alone in patients with recurrent EOC by using real-world data from an Asian population.

## Materials and Methods

### Study Population and Data Collection

**Figure 1:**
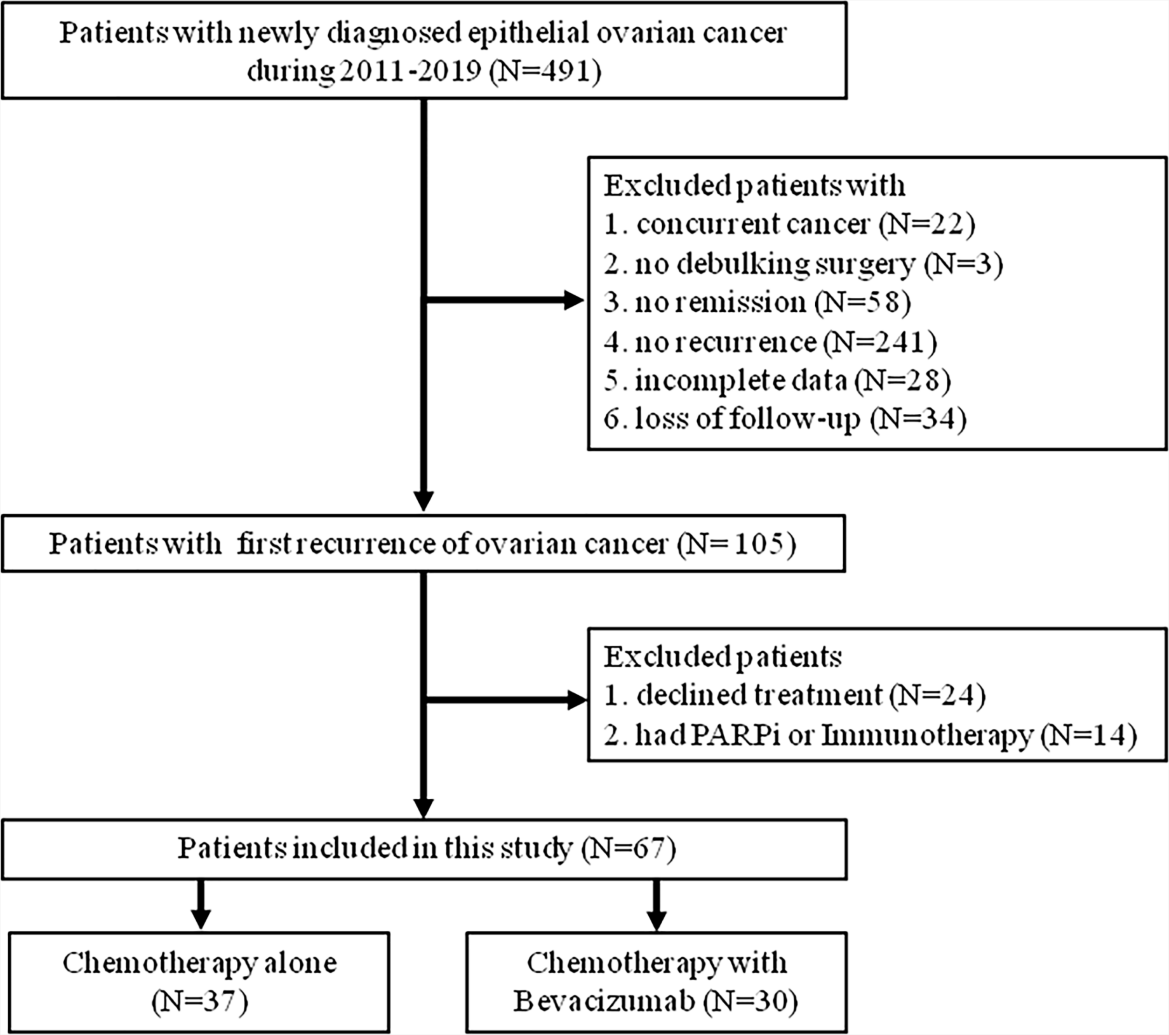
Study flow chart for population selection.

We conducted a retrospective cohort study by using data from a tertiary medical center in central Taiwan. The study protocol was approved by our institutional review board (CE21233B). We identified patients who were newly diagnosed as having EOC between 2011 and 2019 (n = 491, **Figure 1**). After excluding patients who had concurrent cancer (n = 22), did not undergo debulking surgery (n = 3), did not achieve disease remission after treatment (n = 58), did not experience disease recurrence (n = 241), had incomplete data (n = 28), and were lost to follow-up (n = 34), we identified 105 patients with surgically and pathologically proved EOC who had the first recurrence of the disease during the study period. Furthermore, we excluded patients who declined treatment (n = 24) and those who received poly (adenosine diphosphate–ribose) polymerase (PARP) inhibitors or immunotherapy (n = 14) for recurrent EOC. Finally, 67 patients with first recurrence of EOC were analyzed in this study, and they were divided into two groups based on whether they received bevacizumab treatment for the recurrent disease (**Figure 1**).

According to international guidelines, we determined chemotherapy regimens after recurrence based on the treatment-free survival durations of the patients. Women who had relapse less than 6 months after the end of front-line therapy were treated with nonplatinum chemotherapy, whereas those who had recurrence after 6 months were treated with the combination of platinum and nonplatinum drugs. Because bevacizumab was not reimbursed by our national health insurance system before May 2020 (13), the treatment doses and cycles of bevacizumab were affected by cost considerations.

Patients’ medical history, medication treatment data, and relevant information were collected from electronic health records. We assessed our patients’ socioeconomic status by ward fee payment (totally covered by the National Health Insurance or private insurance). The status of disease progression and survival was confirmed in April 2021. Disease progression was defined as an increase in the volume of tumors and the presence of new lesions or ascites on abdominal CT scans performed every 3 months. The primary outcome of this study was overall survival (OS) and PFS. OS was defined as the interval from the initiation of second-line treatment for recurrent EOC to the date of mortality or April 2021. PFS was defined as the interval from the initiation of second-line treatment for recurrent EOC to the date of disease progression, patient mortality, or April 2021.

### Statistical Analysis

Differences in baseline characteristics between the two treatment groups were compared using the Mann–Whitney U test and chi-square test for continuous and categorical variables, respectively. The Kaplan–Meier method was used to generate survival curves for OS and PFS. Cox proportional hazard analysis was performed to determine the association of bevacizumab treatment with OS and PFS after adjustment for relevant variables. Subgroup analysis was conducted to determine if a significant variation was present in the aforementioned association. All statistical analyses were performed using the Statistical Package for Social Sciences (IBM SPSS version 22.0; International Business Machines Corp, NY, USA), and a p value of <0.05 was considered statistically significant.

## Results

**Table 1** lists the characteristics of the study patients according to whether they received bevacizumab treatment for recurrent ovarian cancer. Overall, most of the patients had serous ovarian cancer (76.1%) with advanced stage (88.1%, stage III-IV) at diagnosis. Eight patients (11.9%) underwent suboptimal initial debulking surgery, whereas 11 (16.4%) patients received first-line bevacizumab treatment. Most of the patients (89.6%) had a treatment-free interval of ≥6 months. After disease recurrence, 22 (32.8%) patients underwent secondary cytoreductive surgery, with complete gross resection achieved in all patients, except in one patient in the bevacizumab group. All the patients received doublet chemotherapy, mainly with platinum plus paclitaxel (80.6%) or platinum plus pegylated liposomal doxorubicin. The dose of bevacizumab was 7.5 or 15 mg/kg every 3 weeks, and 76% (23/30) of the patients underwent more than 6 cycles of bevacizumab. The patients underwent fewer cycles due to economic reasons rather than adverse effects. No significant between-group differences were noted in these clinical variables except socioeconomic status (ward fee payment, **Table 1**).

**Table 1:** Characteristics of study participants according to Bevacizumab treatment for recurrence.

Variables	Chemotherapy alone	Chemotherapy with Bevacizumab	P
Number of participants	37	30	
Age at diagnosis, years	59.0 (53.0-66.0)	56.5 (47.8-65.3)	0.316
Cell type, n (%)			1.000
Serous	28 (75.7)	23 (76.7)	
Non-serous	9 (24.3)	7 (23.3)	
Stage at diagnosis, n (%)			0.451
Stage I-II	3 (8.1)	5 (16.7)	
Stage III-IV	34 (91.9)	25 (83.3)	
Suboptimal debulking surgery, n (%)	5 (13.5)	3 (10.0)	0.722
Front-line Bevacizumab use, n (%)	7 (18.9)	4 (13.3)	0.742
Treatment free interval, n (%)			0.692
< 6 months	3 (8.1)	4 (13.3)	
≥ 6 months	34 (91.9)	26 (86.7)	
Cytoreductive surgery for recurrence, n (%)	14 (37.8)	8 (26.7)	0.480
Chemotherapy regimen for recurrence, n (%)			0.842
Platinum plus Paclitaxel or PLD	29 (78.4)	25 (83.3)	
Others	8 (21.6)	5 (16.7)	
Cycles of Chemotherapy	6.0 (5.0-9.5)	7.0 (6.0-9.0)	0.692
Cycles of Bevacizumab	0	8.5 (5.5-16.25)	
Socioeconomic status (ward fee payment), n (%)			0.001
Totally covered by National Health Insurance	29 (78.4)	19 (63.3)	
Private insurance	8 (21.6)	11 (36.7)	

Data are presented as median (interquartile range) or n (%). PLD, pegylated liposomal doxorubicin.

**Figure 2:**
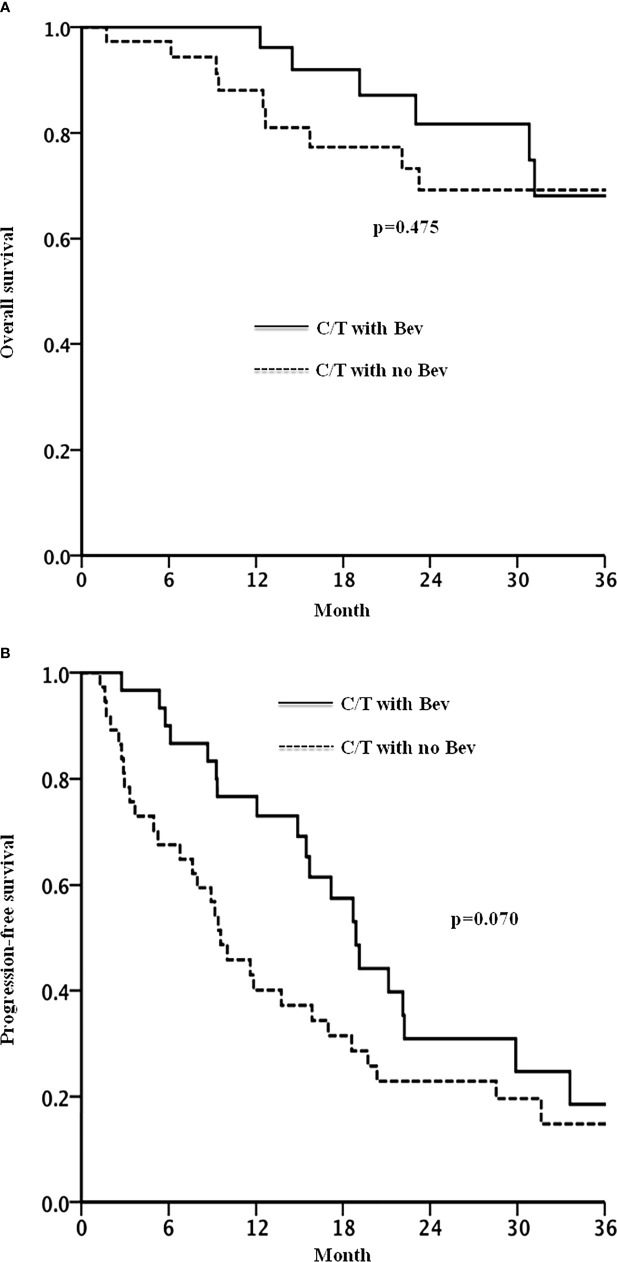
Overall survival **(A)** and progression free survival **(B)** in patients with recurrent ovarian cancer treated with chemotherapy with or without bevacizumab.

**Figure 2** shows the Kaplan–Meier curves of OS and PFS. After a median follow-up of 23 months, 67% of the patients in the bevacizumab group and 81% of the patients in the non-bevacizumab group exhibited disease progression or had died. No significant between-group difference was observed in OS (p = 0.475). The median durations of PFS were 18.9 and 9.6 months, respectively, in the bevacizumab and non-bevacizumab groups, respectively (p = 0.070, **Figure 2**).

**Figure 3:**
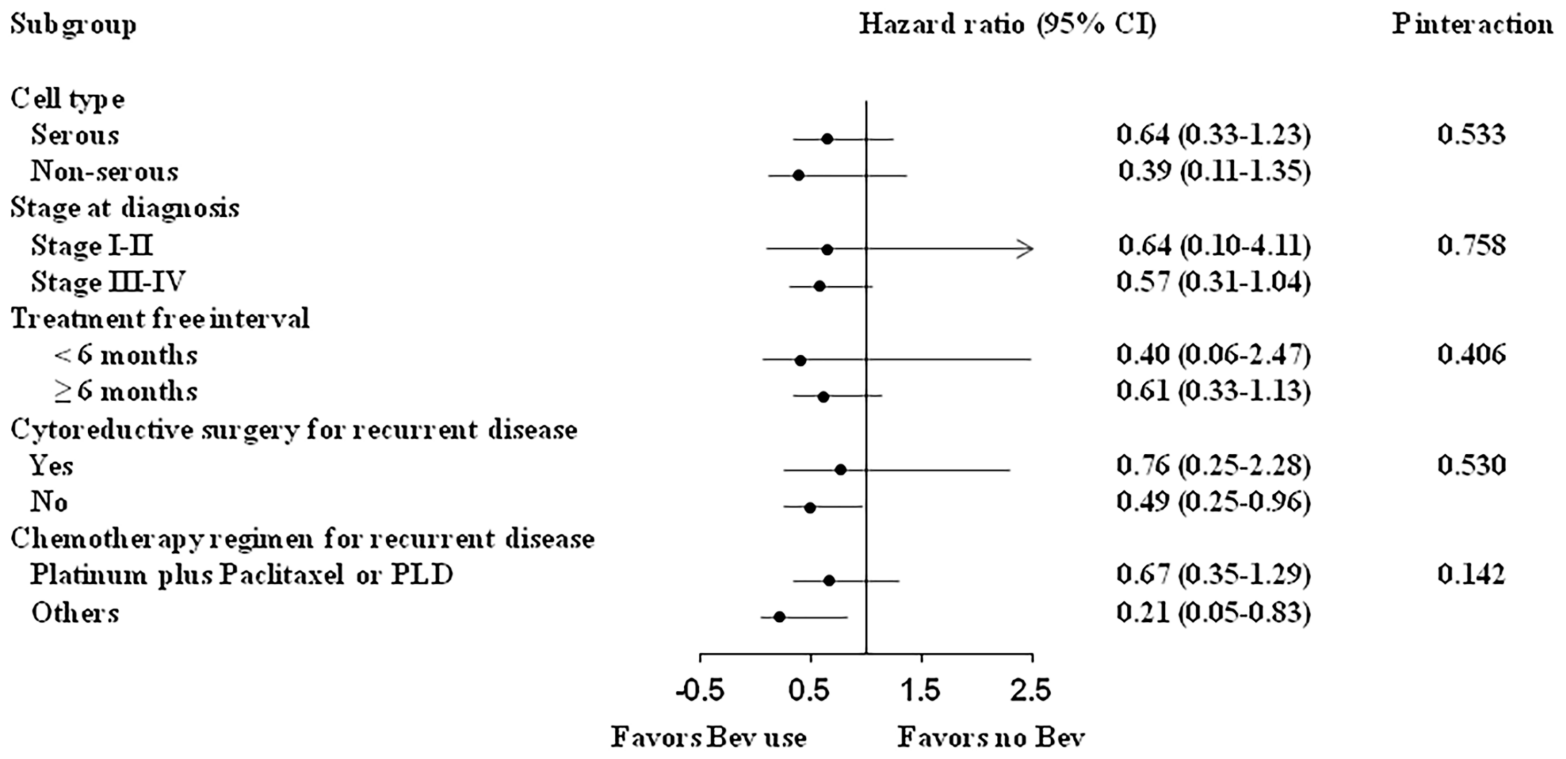
Subgroup analysis of the efficacy of bevacizumab on progression-free survival.

**Table 2** shows the findings of Cox regression analysis for OS and PFS after adjustment for relevant clinical variables. No significant association was observed between bevacizumab treatment and OS. After multivariate adjustment, bevacizumab treatment was associated with a lower risk of disease progression (hazard ratio: 0.33, 95% confidence interval [CI]: 0.13–0.85, p = 0.021). The benefit of bevacizumab treatment for the risk of disease progression was consistent across various subgroups, including subgroups of the cell type, cancer stage at diagnosis, treatment-free interval, secondary cytoreductive surgery, and chemotherapy regimen for recurrent disease (**Figure 3**, all p interaction > 0.05).

**Table 2:** Associations of Bevacizumab treatment for recurrent disease with overall and progression free survival.

	Overall survival		Progression free survival	
Bevacizumab (use vs. no use)	Hazard ratio (95% CI)	P	Hazard ratio (95% CI)	P
Univariate analysis	0.69 (0.24-1.93)	0.478	0.60 (0.34-1.05)	0.074
Multivariate analysis^a^	0.39 (0.08-1.92)	0.244	0.33 (0.13-0.85)	0.021

a Adjusted for stage at diagnosis, histological subtype, suboptimal debulking surgery, front-line Bevacizumab use, cytoreductive surgery, chemotherapy regimen for recurrent disease, and socioeconomic status (ward fee payment).

In terms of the significant adverse effects of bevacizumab treatment, two patients experienced new-onset hypertension, and one patient experienced brain infarction without visible brain metastasis on brain CT. Among patients not treated with bevacizumab, one patient developed rectovaginal fistula after bowel surgery and another patient experienced brain infarction with the evidence of brain metastasis. All other severe adverse effects other than hypertension occurred beyond the second progression.

## Discussion

The results of this study demonstrated a significant improvement in PFS with the addition of bevacizumab to chemotherapy for recurrent EOC (**Table 2**). The improvement in PFS was consistent in the subgroups of different histological types, different disease stages at diagnosis, different treatment-free intervals, those undergoing or not undergoing secondary cytoreductive surgery, and diverse chemotherapy regimens (**Figure 3**). Our findings provided crucial insights into the efficacy of bevacizumab for the treatment of recurrent EOC in the real-world setting.

The improvement in PFS (hazard ratio: 0.33, 95% CI: 0.13–0.85, p = 0.021) in our study was consistent with that observed in previous phase III RCTs. In patients with recurrent platinum-sensitive EOC, the addition of bevacizumab to carboplatin–gemcitabine and carboplatin–paclitaxel significantly improved PFS compared with chemotherapy alone in the Ocean trial (hazard ratio: 0.484, 95% CI: 0.388–0.605, p < 0.001) (7) and GOG0213 (hazard ratio: 0.628, 95% CI: 0.534–0.739, p < 0.001) (14). More recently, the ENGOT-ov 18 trial (15) reported that patients treated with bevacizumab–carboplatin–pegylated liposomal doxorubicin (PLD) had longer PFS than did those treated with different regimens established in the Ocean trial (7). In patients with recurrent platinum-resistant EOC, bevacizumab with a nonplatinum regimen (PLD, paclitaxel, or topotecan) demonstrated a PFS benefit in the Aurelia trial (hazard ratio: 0.48, 95% CI: 0.38–0.6, p < 0.001) (9). On the basis of these trials, the majority of our patients were treated with established treatment combinations. However, most of the RCTs include an almost homogenous group of participants, and patients may receive more focused care and follow-up compared with that typically observed in real-world practice. Moreover, the majority of patients enrolled in the previous trials were from Western countries (94% in OCEAN and 80% in GOG-0213) (7, 14). These factors may limit the extrapolation of results to other populations (16) such as Asian patients with recurrent EOC. Thus, real-world observational studies should be conducted to confirm the effectiveness of treatment under ordinary and often variable conditions.

Real-world data regarding the effectiveness of bevacizumab in Asian patients with recurrent EOC are scant. In a retrospective cohort study (17), the use of bevacizumab in addition to front-line chemotherapy was associated with an improvement in PFS and OS in patients with ovarian, tubal, and peritoneal cancer compared with chemotherapy alone. However, no data on the use of bevacizumab in the recurrent setting are available. In another cohort study, the outcomes of patients with recurrent EOC treated with bevacizumab were reported. However, no “control group” (patients without bevacizumab treatment) was included in that study. Therefore, the efficacy of bevacizumab in the setting of recurrent EOC should be explored. To our knowledge, this is the first real-world study exploring the benefit of bevacizumab treatment for recurrent EOC.

The prolonged PFS in our treatment group (median: 18.9 vs. 9.6 months, difference: 9.3 months) was longer than that reported in previous RCTs (difference: 3–4 months) (7, 14, 18). Uncertain problems regarding the treatment for recurrent ovarian cancer still exist, including those related to the optimal cycles of chemotherapy, combinations of chemotherapy, and dosage of bevacizumab. The cycles of chemotherapy were limited to 6–8 in GOG0213 (14) and 6 in the AGO-OVAR2.5 trial (15). By contrast, patients in real-world practice might receive more cycles of chemotherapy until no measurable disease is observed, tumor markers return to normal levels, or intolerable side effects are experienced. Overall, 40% of our patients received more than six cycles of chemotherapy. Whether more cycles of chemotherapy lead to more favorable outcomes for patients with recurrent EOC warrants further investigation. In addition, we had a higher proportion of patients undergoing secondary cytoreductive surgery (33% in our study) compared with that in the Ocean trial (11%) (7) and GOG 0213 (16%) (14), and almost all the patients had no visible tumor after surgery, which may have contributed to more favorable outcomes in our study.

In our study, treatment with bevacizumab reduced the risk of disease progression in the patients who did not undergo secondary cytoreductive surgery (hazard ratio: 0.49, 95% CI: 0.25–0.96, **Figure 3**) with a nonsignificant between-group difference (in patients who underwent secondary cytoreductive surgery, hazard ratio: 0.76, 95% CI: 0.25–2.28, p interaction=0.530). Theoretically, secondary surgery can act as a drug booster that enhances drug penetration and helps to overcome drug resistance in patients with poorly vascularized tumors. However, secondary surgery poses a risk of surgical complications for patients, and it might postpone bevacizumab treatment and increase its toxicity (such as fistula formation or wound dehiscence). The role of secondary cytoreductive surgery became controversial after the GOG0213 trial; this RCT that included 84% of the patients receiving bevacizumab reported that secondary surgery followed by chemotherapy did not significantly prolong survival compared with no surgery (14). Recently, the SOC-1 trial (19), an RCT conducted in China, demonstrated that secondary cytoreductive surgery could extend PFS to nearly 5 months. The findings are similar to those of the DESKTOPIII trial (20). Notably, the proportion of bevacizumab use was low in these two studies. Based on the aforementioned results, the PFS benefit of bevacizumab and secondary cytoreductive surgery might not be additive. Patients may have various presentations of tumor relapse (different recurrent anatomic sites and lymph nodes) and status of BRCA gene. These factors may also impact patients’ outcomes after secondary cytoreductive surgery, as reported in previous studies (21, 22). Furthermore, new drugs are available (such as PARP inhibitor) for treatment of recurrent EOC. The role of surgical approach should be reconsidered, and individualized treatment is needed (21, 22). Therefore, real-world data on various study populations as a reference for treatment decision are clinically relevant. Considering that patients who are not a candidate for secondary surgery usually have larger tumor burden, extensive metastasis, or poorer prognosis compared with surgical candidates, treatment with bevacizumab in addition to chemotherapy may be an optimal choice for these patients.

This study has several limitations. First, the treatment dose of bevacizumab in our patients was lower than that administered in previous RCTs (7, 9, 14) likely due to financial reasons. We assessed and adjusted socioeconomic status (ward fee payment by National Health Insurance or private insurance) (23, 24), and the effect of bevacizumab on PFS was independent of this factor. More than half of our patients received a treatment dose of 7.5 mg/kg every 3 weeks compared with 15 mg/kg every 3 weeks in previous trials (7, 9, 14). Considering this limitation, bevacizumab treatment significantly improved PFS in our patients. Second, the number of the patients included in our study was small. Finally, because this is not a randomized study, some confounders might exist. Hence, our findings should be confirmed in another study including a larger number of patients.

In conclusion, we demonstrated that treatment with bevacizumab in addition to commonly used chemotherapy significantly prolonged the PFS of patients with recurrent EOC in the real-world setting.

## Data Availability Statement

The raw data supporting the conclusions of this article will be made available by the authors, without undue reservation.

## Ethics Statement 

The studies involving human participants were reviewed and approved by Institutional Review Board of Taichung Veterans General Hospital. Written informed consent for participation was not required for this study in accordance with the national legislation and the institutional requirements.

## Author Contributions

J-NH and C-HL were accountable for conception and design of the study. J-NH, C-HL, S-TH, LS, S-FH, and C-KL performed acquisition of data. J-NH, Y-HS, and J-SW performed the statistical analysis. J-NH, Y-HS, C-HL, J-SW, and M-JC helped interpret the results and wrote the original draft of the manuscript. All authors were involved in the supervision and revision of the manuscript. All authors contributed to the article and approved the submitted version.

## Conflict of Interest

The authors declare that the research was conducted in the absence of any commercial or financial relationships that could be construed as a potential conflict of interest.

## Publisher’s Note

All claims expressed in this article are solely those of the authors and do not necessarily represent those of their affiliated organizations, or those of the publisher, the editors and the reviewers. Any product that may be evaluated in this article, or claim that may be made by its manufacturer, is not guaranteed or endorsed by the publisher.
